# Affimers targeting proteins in the cardiomyocyte Z-disc: Novel tools that improve imaging of heart tissue

**DOI:** 10.3389/fcvm.2023.1094563

**Published:** 2023-02-14

**Authors:** Francine Parker, Anna A. S. Tang, Brendan Rogers, Glenn Carrington, Cris dos Remedios, Amy Li, Darren Tomlinson, Michelle Peckham

**Affiliations:** ^1^School of Molecular and Cellular Biology, Faculty of Biological Sciences, University of Leeds, Leeds, United Kingdom; ^2^Mechanobiology Laboratory, Victor Chang Cardiac Research Institute, Darlinghurst, NSW, Australia; ^3^Sydney Heart Bank, The University of Sydney, Sydney, NSW, Australia; ^4^Department of Pharmacy & Biomedical Sciences, La Trobe University, Bendigo, VIC, Australia; ^5^Centre for Healthy Futures, Torrens University Australia, Surrey Hills, NSW, Australia

**Keywords:** Dilated Cardiomyopathy, Affimer, Z-disc, intercalated disc, fluorescence microscopy, cardiac actinin, ZASP and the N-terminal region of titin (TTN)

## Abstract

Dilated Cardiomyopathy is a common form of heart failure. Determining how this disease affects the structure and organization of cardiomyocytes in the human heart is important in understanding how the heart becomes less effective at contraction. Here we isolated and characterised Affimers (small non-antibody binding proteins) to Z-disc proteins ACTN2 (α-actinin-2), ZASP (also known as LIM domain binding protein 3 or LDB3) and the N-terminal region of the giant protein titin (TTN Z1-Z2). These proteins are known to localise in both the sarcomere Z-discs and the transitional junctions, found close to the intercalated discs that connect adjacent cardiomyocytes. We use cryosections of left ventricles from two patients diagnosed with end-stage Dilated Cardiomyopathy who underwent Orthotopic Heart Transplantation and were whole genome sequenced. We describe how Affimers substantially improve the resolution achieved by confocal and STED microscopy compared to conventional antibodies. We quantified the expression of ACTN2, ZASP and TTN proteins in two patients with dilated cardiomyopathy and compared them with a sex- and age-matched healthy donor. The small size of the Affimer reagents, combined with a small linkage error (the distance from the epitope to the dye label covalently bound to the Affimer) revealed new structural details in Z-discs and intercalated discs in the failing samples. Affimers are thus useful for analysis of changes to cardiomyocyte structure and organisation in diseased hearts.

## 1. Introduction

Dilated Cardiomyopathy (DCM) is a major cause of heart failure worldwide. It has a prevalence of between 1 in 250 to 1 in 400 people (1, 2) and is the leading cause of Orthotopic Heart Transplantation (OHT). is characterised as systolic dysfunction and dilation usually of the left ventricle (LV). It is commonly associated with arrythmias and sudden death (3). In the US the prevalence of familial dilated cardiomyopathy (FDCM) was recently reported at 29.7% (4) but this figure may increase with time. A mutation in cardiac actin (ACTC) was the first to be identified as a possible cause of DCM (5) followed by reports of mutations in cardiac myosin heavy chain (MYH6 and MYH7), troponin T, troponin I and α-tropomyosin (6). Genetic variants in *MYH7* are reported to be the third most common cause of DCM (7), about 10% of all cases. The clinical characteristics for these were recently comprehensively evaluated (8).

Since the first report identifying mutations in TTN as a cause of DCM (9), we now know that TTN truncating mutations (TTNtv) account for about 25% of all familial DCMs (10). Titin is the largest known protein with a molecular weight of ∼3 MDa and a length > 1 μm (11). It spans from the Z-disc (N-terminus) in striated muscle to the central M-band (C-terminus) of striated muscle sarcomeres and is thought to be a key regulator of sarcomere assembly and function (12, 13). The central A-band region is primarily composed of repeating immunoglobulin (Ig) and fibonectin-3-like (Fn-3) domains that predominantly interact with myosin and myosin-binding protein C in the A band. This region of titin is thought to act as a molecular “ruler,” regulating the formation, length and position of the myosin-containing thick filament (11). Its huge size (363 coding exons) and complexity accounts for alternative splicing that results in at least three different isoforms in cardiac muscle (14). TTNvs were only recently identified as a major cause of disease (15, 16).

TTNtvs result in premature stop codons, splice variants and frameshift mutations. TTNtvs are more likely to occur some distance from the N-terminus of the protein (17). They are most common in the A-band region of titin, in both N2A and N2B isoforms and are largely absent from Z-disk and M-band regions (16–18). Variants in the A-band region of titin and are the most pathogenic (18). iPSCs and CRISPR studies have been used to evaluate the effects of these mutations in humans (19).

Here, our main objective was to evaluate the usefulness of using novel, antigen binding proteins called Affimers [originally termed Adhirons (20)] in determining the overall organisation of sarcomeric proteins in frozen sections of DCM tissue from the Sydney heart bank. Affimers are much smaller than antibodies, with a molecular mass of 10–12 kDa and dimensions of ∼2–3 nm (21–23). They are formed of a scaffold consisting of a consensus plant phytocystatin protein sequence, have been engineered to be highly soluble and to have high thermal stability. The binding interface is provided by two regions of variable sequence, approximately 9 residues in length. Affimers to proteins, or protein domains of interest are isolated by screening a phage display Affimer library, in which the amino acids in the regions of variable sequence have been randomised. The ability of the isolated Affimers to bind to their targets are then confirmed by phage ELISA. Each Affimer is then sequenced, and approximately 10 Affimers are then taken forward for further testing. The sequences are subcloned into bacterial expression vectors, to introduce a His tag for purification, and, in our case, a single unique N- or C-terminal cysteine, to enable direct fluorescent dye labelling. Purified dye labelled Affimers are then tested for their ability to label structures of interest efficiently and specifically, with low background. The best performing Affimer is then used in subsequent experiments.

In this new work, we report that Affimers work better than antibodies in labelling samples of control and DCM tissue from the Sydney Heart Bank (24). We tested Affimers to the cardiac isoform of α-actinin-2 (25), ZASP (Cypher/Oracle/Enigma: a PDZ-LIM protein) and the Z1Z2 repeats of titin. Samples of DCM tissue from this heart bank have already been shown to be useful in evaluating the effects of mutations on the contractile properties of myofibrils from DCM hearts (26, 27), with some analysis on the morphology of this tissue (28). The small size of the Affimer reagents enhances their ability to penetrate tissue sections and improves their ability to identify regions within the dense cytoskeleton, compared to conventional antibodies or even their small (Fab) fractions. To demonstrate their efficacy, we focused on heart tissue samples derived from two different patients [see (26)], both of which have a single TTNtv frameshift variant implicated in FDCM (p.R23464Tfs*41) and compared these to samples from an age and sex-matched control.

## 2. Results

### 2.1. Affimers to Z-disc protein domains

We isolated Affimers to three Z-disc proteins, by screening a phage display library against the calponin homology (CH) domains of α-actinin-2 (ACTN2) the Z1Z2 repeats of titin, and full length ZASP (Isoform 2 of LIM domain-binding protein 3, also known as cypher). ACTN2 crosslinks actin filaments within the Z-disc. The Z1Z2 repeats of titin are formed of Ig domains and are found within the N-terminal region of titin, located in the Z-disc (29). ZASP (Z-disc alternatively spliced PDZ-motif) is a member of the ALP/Enigma family (30), forms a multiprotein complex ACTN2 and is implicated in signalling (31). All the Affimers, confirmed to bind to their protein targets by phage ELISA (data not shown), were subcloned into bacterial expression vectors, expressed, purified, dye labelled and tested for their ability to label Z-discs. A single Affimer for each target, that labelled Z-discs specifically and showed low background staining was then taken forward for further analysis.

Next, we were interested to determine if Alphafold modelling could be useful in predicting the site of interaction between the Affimer and its target. We already know the site of interaction for the ACTN Affimer as we previously solved a crystal structure of the Affimer bound to the CH domains of ACTN2 [(PDB: 6SWT (25)] and see Supplementary Figure 1A). However, we have not yet obtained crystal structures for the ZASP and titin Z1Z2 Affimers, and Alphafold could provide a good fast alternative approach to crystallisation, to determine the site of interaction.

First, we compared the structures predicted by Alphafold modelling with our published ACTN2-CH domain-Affimer crystal structure (PDB: 6SWT) to determine the efficacy of Alphafold in predicting the mode of binding (Supplementary Figure 1). Alphafold did correctly predict the structures of the isolated CH domain structure of ACTN2 (RMSD ∼0.29Å) and the Affimer (RMSD ∼0.6Å) with a high degree of accuracy (Supplementary Figures 1A, B, 2). For the CH-domain-Affimer complex, two of the five Alphafold predictions show a similar interaction of the Affimer with the ACTN2 CH domain to that found in the crystal structure, in which variable loop-1 of the Affimer interacts with a loop within CH domain 2 (Supplementary Figures 1A, B). This suggests that Alphafold can, with some degree of certainty, predict the epitope on the protein of interest that the Affimer is recognising.

Next, we used Alphafold modelling for the Affimer-Z1Z2 complex and the Affimer-ZASP complexes. From the results, we speculate that the Z1Z2 Affimer likely recognises the C-terminus of Z1Z2 (Z2 domain; 3 hits) or the unstructured loop connecting the 2 domains (2 hits) (Supplementary Figures 1C, 2). However, this approach was less successful for ZASP, which is predicted to be largely disordered, with the exception of the first 84 residues that form a PDZ domain, a site of interaction for ACTN2 (32). Not surprisingly, the confidence in ZASP structural prediction is considerably lower, with the regions connecting between the relatively well-structured N- (∼1–100aa) and C- (∼420–617aa) regions showing per residue confidence (pLDDT) scores below 50% confidence (Supplementary Figure 2). Omitting regions with a confidence score of less than 30% suggests that the ZASP Affimer recognises an epitope at the very C-terminus of ZASP (Supplementary Figure 1D). Thus, Alphafold could be useful for predicting sites of interaction in the future, but the degree of confidence in these results is variable. However, knowing the precise site of interaction is not essential to using the Affimers in downstream applications, such as staining of tissue samples.

### 2.2. Affimer tissue penetration is improved compared to antibodies

**FIGURE 1:**
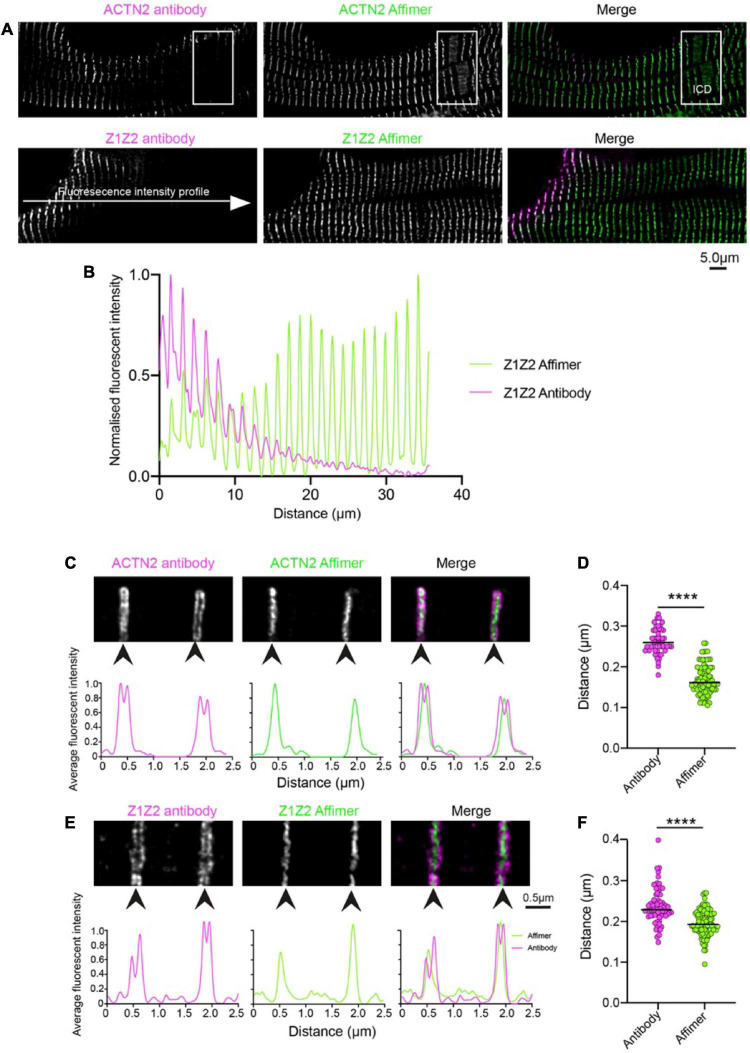
Comparison of staining heart sections using antibodies and Affimers to ACTN2 and titin Z1Z2 repeats. **(A)** Example confocal image of a region of a donor heart section stained using a primary antibody to ACTN2 or the Z1Z2 repeat of titin combined with a secondary fluorescent antibody, and with a dye-labelled Affimer. The boxed region shows the position of the ICD: intercalated disc. **(B)** Example of fluorescence intensity (normalized) for a line profile drawn across the cell for the ACTN2 antibody (magenta) and Affimer (green). Example 2D-STED images for Z-discs stained using the ACTN2 primary and secondary antibody combination and the dye-labelled ACTN2 Affimer **(C)** and the titin Z1Z2 antibody combination and Z1Z2 Affimer **(E)** are shown together with the associated profile plots for the labelling intensity across the Z-disc structures. **(D,F)** Measurements of the Z-disc widths for multiple Z-discs from sections labelled with the antibody combination and Affimers, using either the antibody images or the Affimer images. ****p < 0.0001.

To determine the specificity of Affimers to effectively label protein structures in human heart sections, we compared the staining results using Affimers and antibodies to ACTN2 and to the Z1Z2 repeats of titin imaged by confocal (Antibodies to ZASP were not available to us). The resulting images showed that fluorescent labelling of the heart sections was much more uniform across the whole section for ACTN2 and Z1Z2 Affimers compared to that for anti-ACTN2 and Z1Z2 antibodies (Figure 1A). The fluorescence intensity across the section was highly variable, when the sections were labelled with antibodies against ACTN2 and Z1Z2, followed by secondary fluorescent antibodies, with levels of labelling higher toward the edge, or less dense parts, of the section. In contrast, the fluorescence intensity across the section was much more uniform, when the sections were labelled with Affimers (Figure 1B). In addition, the transitional zones close to the intercalated discs (ICD) were labelled well by the Affimer but labelling by the antibody was less uniform (Figure 1A: boxed region).

Super-resolution (STED) microscopy was used to further compare the ability of Affimers and antibodies to label the Z-disc. The xy-resolution of confocal microscopy (∼200 nm, or ∼170 nm in Airyscan mode) is not sufficient to resolve any detail within the Z-disc [approximately 100–140 nm in cardiac tissue (33)]. The xy-resolution for 2D-STED (stimulated emission depletion) microscopy is approximately 50nm and can resolve some structure within the Z-disc.

STED imaging of the same tissue sections used for confocal microscopy (Figure 1A) further demonstrates that the small size of the Affimers allows them to better penetrate the Z-disc structure and label ACTN2 and Z1Z2 repeats of titin, which should be distributed through the Z-disc, compared to antibodies. ACTN2 and Z1Z2 antibodies labelled the edges of Z-discs but were mostly absent from the central region of the Z-disc (Figures 1C, E). This variation in staining is demonstrated by intensity profile plots across the Z-disc, which revealed two peaks for the antibody labelling at the edges of the Z-disc (Figures 1C, E). In contrast, the Affimers labelled the Z-disc uniformly throughout (Figures 1C, E). It is worth noting here that the Z1Z2 repeats were first reported to be located in the central region of the Z-disc (29) whereas a later study using the Z1Z2 antibody used here showed that they were located toward the edge of the Z-disc (34). Our work with the Affimer suggests that the first report is likely to be correct.

The small size and direct labelling of Affimers puts the dye label very close to the epitope that the Affimer recognizes (∼4 nm). In contrast, the combination of primary and secondary antibodies typically puts the dye label much further away (∼30 nm). The average width of the Z-disc measured from deconvolved STED images, using specimens labelled with both antibody and Affimer, was larger (262 and 238 nm for ACTN2 and Z1Z2 respectively) using the antibody labelling than using the Affimer labelling (163 and 197 nm). The values measured for the Affimers are closer to the 130 nm width measured for the Z-disc in vertebrate cardiac muscle from EM data (35) (Figures 1D, F).

### 2.3. Affimers detect molecular changes in sarcomeres from DCM patient samples. Z-discs are thicker and sarcomeres are shorter

Having confirmed that the ACTN2 and Z1Z2 Affimers outperform antibodies in labelling these proteins within the Z-disc, we then used these 2 Affimers and one additional Affimer isolated to ZASP to stain the Z-discs in control samples (age, sex matched) and in two heart samples from two different DCM patients (DCM1 and DCM2) sharing the same A-band titin mutation. All the samples were labelled with the ACTN2 Affimer and co-labelled with either ZASP or Z1Z2 Affimers.

**FIGURE 2:**
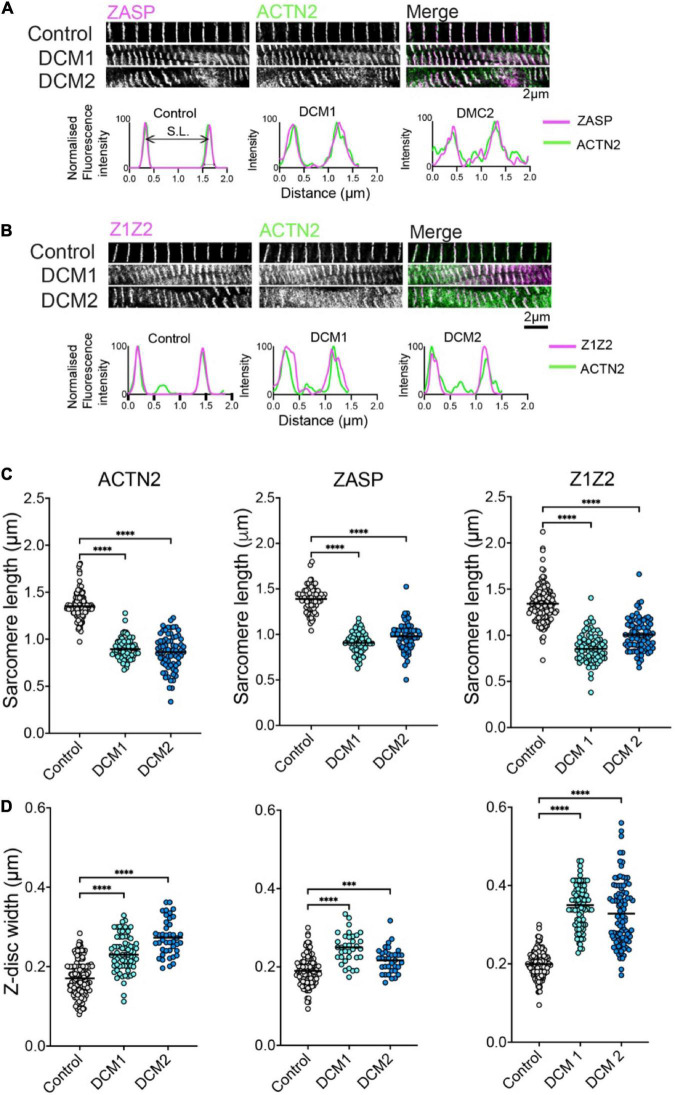
ZASP, ACTN2 and titin Z1Z2 Z-disc labelling using Affimer combinations and analysis. **(A)** Example 2D-STED images of heart tissue sections from control (donor tissue) and two independent DCM patients with the same TTN mutation, co-stained using an Affimer to ZASP (magenta in merged image) and ACTN2 (green in merged image). Example line profiles for ZASP and ACTN2 across two a single sarcomere, including both Z-discs at either side are shown below. These profiles were used to estimate Z-disc width and sarcomere length. **(B)** Example images of heart tissue sections from control and DCM patients stained using an Affimer to titin Z1Z2 (Magenta in merged image) and ACTN2 (green in merged image). Example line profile plots are shown below. **(C)** Measurements of Z-disc width for control and DCM patients. **(D)** Measurements of sarcomere lengths for control and DCM patients. Measurements were made from at least 50 sarcomeres from three independently stained heart tissue sections for controls and for each of the DCM patient tissue. ^***^p < 0.001, ^****^p < 0.0001 for comparisons of DCM1 and DCM2 to control.

In donors and DCM heart samples, all three Affimers labelled the Z-disc well. The labelling showed the characteristic striated pattern expected for Z-discs in cardiomyocytes in both control and DCM samples (Figure 2). In DCM samples, the myofibrils were less well organized with evidence of myofibrillar disarray and misalignment (Figures 2A, B). In addition, the width of the Z-disc was increased compared to controls, and the spacing between Z-discs (sarcomere length) decreased. To quantify this, we measured the Z-disc widths and sarcomere length for control and DCM patient tissue using deconvolved 2D-STED images of Z-discs labelled by each of the three Affimers (Figures 2A, B). Sarcomere lengths (distance between Z-discs) were significantly decreased (Figure 2C) and Z-disc widths significantly increased (Figure 2D) in heart samples from the two DCM patients compared to controls.

The Z-disc widths measured for ACTN2 and Z1Z2 in this second dataset for samples co-stained with Affimers are consistent with the measurements for of Z-disc widths measured for the Affimers in the first independent dataset in which samples were co-stained with antibodies and Affimers (Figures 1D, F). Interestingly, the Z-disc width measured for Z1Z2 is increased compared to that measured for ACTN2 in both. Z-disc width measured for Z1Z2 (200 ± 33 nm, mean ± SD) is significantly higher than that measured for ACTN2 (170 ± 0.040 nm: mean ± SD) and may reflect a wider distribution of Z1Z2 titin epitopes across the Z-disc, compared to ACTN2.

### 2.4. Z-disc Affimers label the edges of intercalated discs

Cardiomyocytes connect to each other at the intercalated discs, structures that enable communication between cardiomyocytes. The plasma membrane in this region is highly folded, and a transitional zone has been reported in which Z-disc proteins (ACTN, titin) assemble into a structure at the position in which the final Z-disc of the muscle sarcomere would be expected to be found (36). A titin antibody (T12) that labels a region of titin just outside of the Z-disc showed a doublet distribution either side of the intercalated disc (36). The thin filaments have been suggested to pass through this transitional zone and insert into the adherens junction in the intercalated disc, to enable effective structural integration of the myofibrils at this junction (36).

**FIGURE 3:**
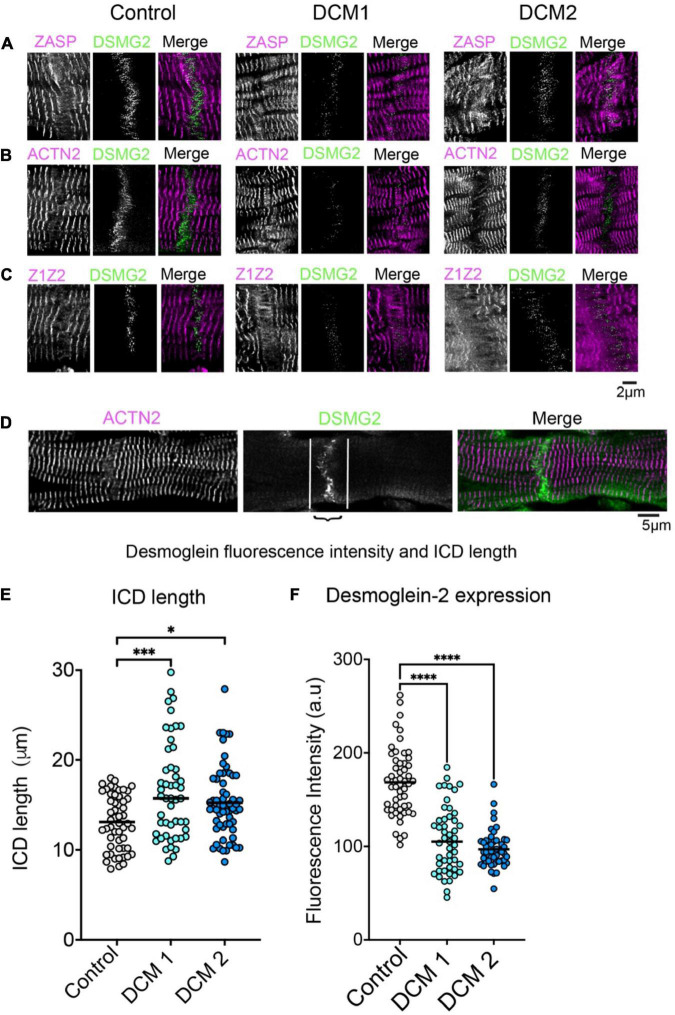
Labelling of intercalated discs (ICDs) between cardiomyocytes in heart tissue sections with Z-disc Affimers and desmoglein. Panels **(A–C)** are examples of labelling for ZASP **(A)**, ACTN2 **(B)** and titin Z1Z2 **(C)** for control (normal donor heart) and DCM heart tissue sections. Panel **(D)** shows desmoglein staining for a single ICD in more detail to show the region of interest used to estimate labelling intensity for desmoglein (DSMG2) in the ICD. Panel **(E)** shows the average length of the ICD and **(F)** the fluorescence labelling intensity for desmogelin staining, for controls and DCM patients. A minimum of 50 ICDs were analysed for three independently stained heart tissue sections. *p < 0.05, ^***^p < 0.001, ^****^p < 0.0001 for comparisons of DCM 1 and DCM2 to control.

Focusing on Affimer labelling at the intercalated disc, which was identified by antibody labelling for desmoglein-2, revealed that ZASP, ACTN2 and Z1Z2 Affimers all label a structure close to the ICD (Figures 3A–C, WT) likely to be the transitional zone. We also observed that there was some labelling within the ICD, at right-angles to, and crossing the junction, possibly structures within the membrane. The intensity of ICD labelling using an antibody to desmoglein-2 was significantly lower in heart tissue samples from both DCM patients (Figures 3B, C, F). In addition, Affimer staining in this region was much less ordered, especially in DCM2 compared to control samples (Figure 3F). Due to the highly disordered nature of the Affimer staining here, it was not possible to quantify the expression levels of the Z-disc proteins. Cardiomyocyte width estimated from the length of desmoglein-2 labelling across the end of the cardiomyocyte within the ICD was slightly increased in DCM samples (Figure 3E).

## 3. Discussion

Here we report that Affimers to ACTN2, ZASP and titin (Z1Z2) are excellent tools for labelling the cardiac cytoskeleton. The Affimers label structures across the tissue sections with better uniformity, and better penetration of the compact Z-disc compared to traditional primary and secondary antibody combinations. The Z-disc widths measured using Affimers are closely aligned with values obtained by electron microscopy. They demonstrate that the Z-disc thickness increases in DCM patients LV tissue compared to controls, an increase that was consistently observed for the three Affimers, while the sarcomere length was decreased. All three Affimers labelled the transition zone in the intercalated disc, with some Affimer labelling within the disc. Both DCM patients showed alterations to the structure of the intercalated disc.

Z-discs are narrow structures that vary from about 100–140 nm in width in cardiomyocytes to as little as ∼30 nm in width in skeletal muscle (33). They are important structural and signalling centres and contain many different proteins. Accurately measuring the width of Z-discs using the traditional primary and secondary antibody combination is challenging due to their large size which limits penetration of the antibodies into the Z-disc structure, and the positioning of the fluorophore ∼30nm away from the target epitope. Here, the combination of STED microscopy and Affimers allowed more accurate measurements of Z-disc width, more consistent with that measured by electron microscopy. The Z-disc width increased for both DCM patients, which both harbour the same TTNtv.

Titin is a key molecule that is assembled into the Z-disc via its N-terminal domains and into the M-band via its C-terminal domains, spanning half a muscle sarcomere (14). In patients with TTNtv variants, not only are levels of wild-type titin reduced, but truncated titin isoforms are also present at least for TTNtv variants, where the truncation is relatively distal to the N-terminus. The p.R23464Tfs*41 truncation is relatively distal to the N-terminus and can integrate into the muscle sarcomere (reviewed in (37–39). The increased Z-disc width is consistent with the idea that a full-length intact titin molecule is required to transmit and buffer force generated by myosin during contraction (40) and that shorter variants of TTN such as the TTNtv variants likely result in disordered Z-discs.

Intercalated discs are critical for attachment of cardiomyocytes cell-cell signalling and communication. Here we found ACTN2, ZASP and titin Z1Z2 are all localised to the transition zone of the intercalated discs, consistent with earlier findings (36, 41, 42). We also show evidence of staining within the intercalated discs, not seen before, which highlights their ability to reveal new structures. This transitional zone has been suggested to act as a site for generating a new Z-disc complex and for sarcomere addition (41). Alterations to the structural integrity of the intercalated disc has previously been suggested to be important in cardiomyopathies (43, 44) and mutations in intercalated disc proteins also result in cardiomyopathies (45). More work is needed to understand how TTNtv variants can affect the structure and organisation of the intercalated discs.

In conclusion, we have shown that Affimers are excellent tools in analysing heart samples. Importantly, Affimers are easy to make, stable, and simple to use in labelling proteins in heart tissue sections. They outperform antibodies in imaging these sections, through better penetration, and their small size improves resolution. A simple one-step staining procedure makes staining easier, and overcomes any problems caused by using traditional labelled secondary antibodies. Specifically, we have demonstrated that Affimers can be used to characterise disease phenotype and reveal alterations to the structure and organisation of cardiomyocytes in patients with cardiomyopathies. If used in the clinic, they could be a useful tool to confirm the phenotype and help to diagnose DCM, if, as we anticipate, DCM generally leads to a widening of the Z-disc.

## 4. Materials and methods

### 4.1. Donor tissue and ethical approval

Anonymised tissue samples from explanted and donor hearts in the Sydney Heart Bank were used in this study (Table 1). Two patients with a diagnosis of familial DCM requiring a heart transplant at a young age, both with the same TTN frameshift mutation (SHB code 4.100 (Male, 22years) and 4.125 (Male, 37 years) were selected together with an age/sex matched non-cardiac death donor (SHB code 6.038 (Male 37 years, non-cardiac death) as a control. Patients were consented under ethical approvals obtained from St Vincent’s Hospital, Darlinghurst (HREC #H91/048/1a), the University of Sydney (HREC #2016/923). The heart samples for DCM were snap-frozen within 20–30 min of the loss of coronary blood flow. Frozen samples were shipped to the University of Leeds for analysis and stored and processed under ethical approval BIOSCI 17-015.

**TABLE 1:** A summary of the details of the LV samples used in this report.

SHB code	Sex/Age (y)	Diagnosis	LVEF%	NYHA	Mutated gene	LR/RV co-morbidities	Publication
4.100	Male/22	DCM	15–20	IV	TTNtv	No CAD	(26, 48)
4.125	Male/36	DCM	15	IV	TTNtv	Dilated LV-RV. LAD 50% occluded	(27, 49)
6.038	Male/25	Donor	50+	NA	None	No CAD (cervical dislocation)	(50, 51)

The Sydney Heart Bank (SHB) code is an anonymised patient label. Patients were diagnosed with familial Dilated Cardiomyopathy (DCM) with both carrying a truncating mutation of the giant TTNtv gene. The clinical records for patient 4.100 included an unsupported note suggesting it may be “post-viral” but otherwise he exhibited no co-morbidities. The Pathology report for patient 4.125 confirmed he had 50% occlusion of the left anterior descending (LAD) artery only. Publications listed support the DCM and donor status of these patients (column 3). SHB: LVEF: left ventricular ejection fraction. NYHA: New York Heart Association classification of heart failure. (IV: class IV – severe).

### 4.2. Affimer screening and expression

The Affimer reagent to α-actinin-2 (ACTN2), isolated against the CH domains from ACTN2, has been described previously (25). Two new Affimer reagents were isolated to the N-terminal region of titin (Z1/Z2: residues 1–200) and to the ZASP (isoform 2: NP-001073583: 1–617 residues). To isolate the Affimers, target protein constructs were expressed and purified using *E. coli*. For Z1/Z2, a codon-optimised Z1/Z2 cDNA construct, cloned into pET28a-SUMO vector with a His tag for affinity purification was expressed in BL21 DE3 cells (Novagen), purified using NiNTA chromatography followed by size exclusion chromatography. The purified protein was then biotinylated before using in the Affimer screen. These two Z1Z2 domains of titin are located within the Z-disc (13). For ZASP, the coding sequence was cloned into a pGEX-Avitag vector (a kind gift from Christian Tiede) in frame with a C-terminal 15 residue Avitag (GLNDIFEAQKIEWHE) and a 6-His N-terminal tag for affinity purification. The protein was biotinylated in *E. Coli*, by co-expressing with pBirA using AVB101 bacterial cells (Avidity). Cells were grown in TYH (Tryptone, Yeast Extract, HEPES) medium supplemented with 0.5% glucose until the OD_600_ reached 0.7. Protein expression and biotinylation was induced by the addition of 1.5 mM IPTG and 50 mM biotin solution (12 mg of d-biotin in 10 ml of 10 mM bicine buffer, pH 8.3) for 3 h. The expressed protein was purified by NiNTA chromatography. All expressed proteins were checked for purity. Western blots with streptavidin-HRP (Sigma) were performed to confirm the purified protein was the correct size and that it was the only protein that was biotinylated. Approximately 1.0 mg/ml of biotinylated protein were used in an Affimer screen as described (23, 25). Following the screen, approximately 8–10 Affimers that bound to the protein of interest, as demonstrated by ELISA were subcloned into pET11a for bacterial expression, using the *Not*I and *Nhe*I restriction sites and a unique cysteine residue was added at the C-termini to allow maleimide conjugation to a fluorescent dye.

### 4.3. Expression, purification, and Affimer labelling

Affimer expression was as described previously (23, 25). Cells from the expression cultures were harvested, pellets were frozen and then thawed on ice and lysed by addition of 1ml lysis Buffer (50 mM NaH_2_PO_4_; 500 mM NaCl; 30 mM Imidazole; 20% Glycerol; pH 7.4, supplemented with 1x HALT protease inhibitor cocktail (Promega), 0.1mg/ml lysozyme (Sigma), 1% Triton X-100 and 10 U/ml DNAse) for one hour on a rotor mixer at room temperature. Low stability *E. coli* proteins were denatured by heating to 50°C for 20 min (Affimers are stable at 50°C) and the insoluble fraction was pelleted at 16,000 g for 20 min. The supernatant was mixed with 300 mL Ni-NTA resin at room temperature for 1 h, washed in wash buffer (50 mM NaH_2_PO_4_; 500 mM NaCl; 20 mM Imidazole; 20% Glycerol; pH 7.4) and then eluted in the same buffer with 300 mM Imidazole. The concentration of Affimer eluted from the column was monitored by absorbance at A_280_ on a NanoDrop spectrophotometer.

Affimer labelling was performed immediately after elution from the Ni-NTA column. Affimers were diluted to 1.0 mg/ml in PBS (phosphate buffered solution) and cysteine activated by mixing with immobilised TCEP (tris(2-carboxyethyl)phosphine) denaturing gel (Thermo Scientific) for 1 h at room temperature. Following a brief centrifugation of 1,000 rpm for 1 min, 130 μl of supernatant was removed and mixed in a 1.5 ml tube with 6 μl of a 2 mM stock maleimide-fluorescent dye (Abberior STAR 580- or STAR 635P-maleimide, Abberior) for 2 h at RT or overnight at 4°C. The reaction was quenched by the addition of 1.3 μl of β-mercaptoethanol for 15 min at room temperature. Labelled Affimers were dialysed against PBS to removed unbound dye (Snakeskin Dialysis Tubing molecular weight cut off 3.5, Pierce) or purified using PD SpinTrap G-25 columns (Cytivia) following manufacturers’ instructions. SDS-PAGE was performed to assess Affimer purity and labelling.

### 4.4. Tissue preparation, staining, and imaging of samples

Each of the 8–10 Affimers, isolated for each of the three protein targets (ACTN2, Z1Z2 and ZASP) were tested for their ability to label Z-discs in heart sections, and from these, the Affimer that gave the best signal, specific Z-disc labelling with low background, was taken forward for the remaining experiments presented here. To prepare sections, frozen left ventricular heart tissue was embedded in O.C.T (optimal cutting temperature) compound and brought up to cryosection temperature of –20°C. 10 μm thick sections were cut using a cryostat (Leica Biosystems) and adhered directly to SuperFrost Plus slides (Fisher Scientific). A PAP pen was used to draw a hydrophobic barrier around the section and the tissue was fixed in 4% paraformaldehyde for 60 min at room temperature before washing three times in PBS containing Tween-20 for 5 min each.

To label sections, the sections were first blocked in in phosphate buffer (PBS) containing 0.5% Triton X-100 and 10% BSA (bovine serum albumin) for 1 h, then incubated with 10 mg/ml Affimer or primary antibody, diluted in blocking buffer, for 1 h at RT or O/N at 4°C. Following washing, in PBS -Tween, samples were either incubated for an hour with secondary antibody diluted in blocking buffer and either washed again or (for Affimer staining only, where a second incubation step is not required) mounted directly by adding a drop of ProLong Gold Antifade (Invitrogen) onto the section, then placing a cleaned glass coverslip [#1.5: (Scientific Laboratory Supplies)] on top of the samples. All the samples were labelled for identification using an alphanumeric code, to avoid bias in imaging and subsequent analysis.

Confocal imaging used an inverted Zeiss LSM880 in Airyscan mode, using the ×40 N.A 1.4 objective lens and the same laser power settings for each sample. STED imaging used an Abberior STEDYCON and a ×100, N.A. 1.4 objective lens, with the depletion laser set for ∼50nm resolution.

In addition to the Affimers, commercial mouse monoclonal anti-actinin antibody EA-53 (Sigma-Aldrich, 1:500: raised to the full length ACTN protein) and a mouse monoclonal antibody to human desmoglein-2 (CCSTEM28; eBiosciences from Thermo Scientific, 1:200) were used followed by secondary anti-mouse StarRed antibodies (Abberior, 1:100). The rabbit polyclonal titin Z1Z2 antibody was generously provided by Bang et al. (14) (raised against the NH2-terminal 195 residues of the human cardiac titin) and used with secondary anti-rabbit Star Red antibodies. Affimers used in these experiments were directly labelled with STAR 580.

### 4.5. Sarcomere analysis

STED images were deconvolved using the deconvolution wizard in Huygens software (SVI, Netherlands). For sarcomere length measurements, straight lines were drawn across a run of 10–20 sarcomeres in ImageJ, beginning and ending at a Z-disc. To estimate the average sarcomere length, the lengths of these lines were measured and divided by the number of sarcomeres (Z-discs). Measurements were repeated for a minimum of 50 times with specimens from 3 separate experiments, using a minimum of 3 sections per control and for each of the two DCM patients. Samples were labelled alpha-numerically to prevent user bias.

Z-disc width, intercalated disc length and diameter of the intracellular storage vesicles were also measured using ImageJ using the deconvolved STED images. A thick line (74pt) was drawn across the structures, and plot profile was used to determine the intensity profile and derive the average widths (or lengths) of the structures. To determine the expression levels of desmoglein-2, the average fluorescent intensity along intercalated discs was measured with ImageJ using Airyscan confocal images where the settings remained constant for each sample. Data was collected from 3 separate experiments using a minimum of 3 sections per control and for each of the two DCM patients. All data was analysed and plotted using Prizm (Graph Pad). Significant changes between samples were tested by Anova with *post hoc* analysis.

### 4.6. Aphafold2

Affimer:protein complexes were generated using the ColabFold implementation of AlphaFold (46, 47). Models of the ACTN:Affimer complex were compared with the published CH domain from actinin in complex with an Affimer crystal structure (6SWT) in Chimera.

## Data availability statement

The original contributions presented in this study are included in the article/Supplementary material, further inquiries can be directed to the corresponding author.

## Ethics statement

Patients were consented under ethical approvals obtained from St. Vincent’s Hospital, Darlinghurst (HREC #H91/048/1a), the University of Sydney (HREC #2016/923). Frozen samples were shipped to the University of Leeds and stored and processed under ethical approval BIOSCI 17-015. The patients/participants provided their written informed consent to participate in this study.

## Author contributions

DT and AT performed and analysed the Affimer screen to proteins supplied by FP and BR. AL provided the samples from SHB. FP prepared and labelled Affimers, performed the staining, imaging, and analysis of the heart sections. MP performed additional imaging. MP and FP designed the experiments and wrote the manuscript. GC performed the Alphafold modelling. CR and AL provided the clinical details of the heart samples. All authors reviewed the final version of the manuscript.
