# Supplementary material for: Affimers targeting proteins in the cardiomyocyte Z-disc: Novel tools that improve imaging of heart tissue
Source: Front Cardiovasc Med. 2023 Feb 14;10:1094563. doi: 10.3389/fcvm.2023.1094563 (PMC9971620; doi:10.3389/fcvm.2023.1094563)
Supplement: Supplementary file 1 [file Data_Sheet_1.docx]

Supplementary Material

## Supplementary Figures


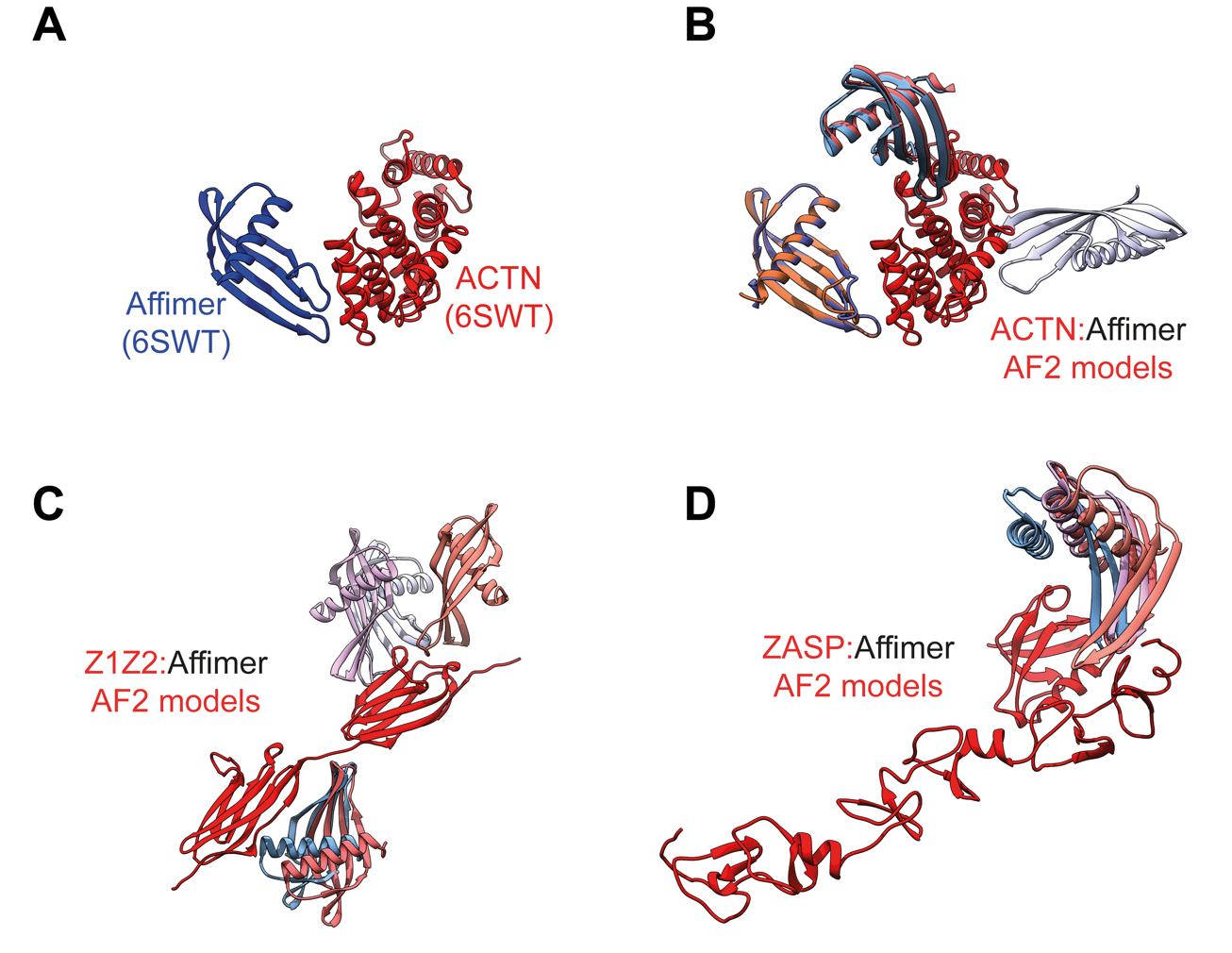


**Supplementary Figure 1.** **Alphafold predictions of Affimer-protein domain complexes for ACTN2, ZASP and titin Z1Z2. A:** CH:Affimer9 complex (6SWT) solved to 1.2 Å. B: Comparison of 5 Affimer:ACTN AF2 complex predictions aligned on the CH domain as in (A). C: Comparison of 5 Affimer:Z1Z2 AF2 complex predictions aligned on the Z1Z2 structure. D: Comparison of 4 Affimer:ZASP AF2 complex predictions aligned on the N- (1-81aa) & C- (550-617aa) domains of ZASP. Structure with pLDDT confidence <50% were omitted from the structure.


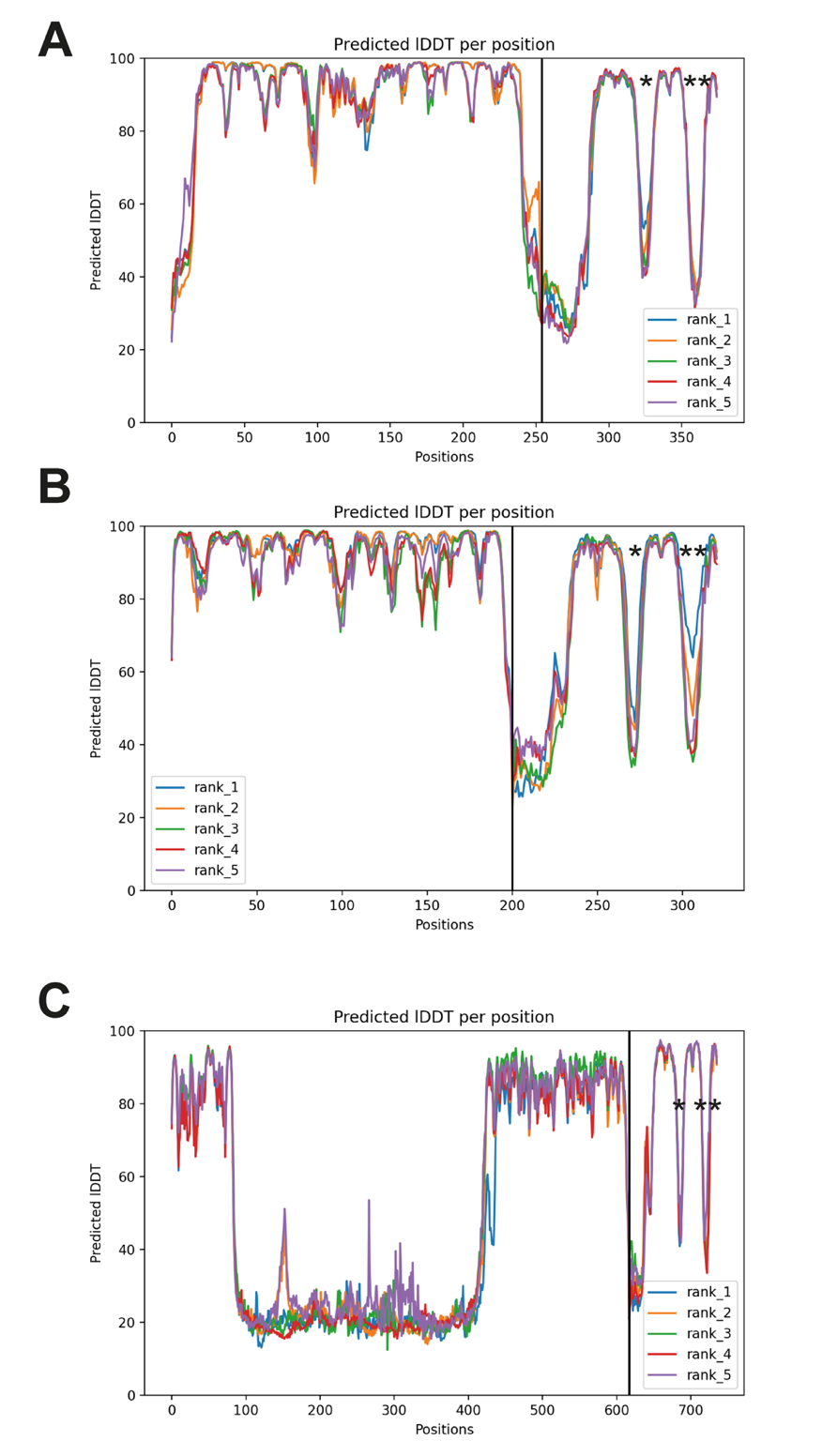


**Supplemental Figure 2**. **Predicted IDDT per residue for 5 models (rank 1 – 5) of ACTN (A), Z1Z2 (B), & ZASP (C) in complex with an Affimer.** The left hand side of each graph corresponds to the target protein, while the right hand side refers to the Affimer. The Affimer binding loops 1 (*) and 2 (**) are shown on each graph. Residues with pLDDT scores <30% were omitted from the structures.
